# Ultra High Field fMRI of Human Superior Colliculi Activity during Affective Visual Processing

**DOI:** 10.1038/s41598-020-57653-z

**Published:** 2020-01-28

**Authors:** Yuxi C. Wang, Marta Bianciardi, Lorena Chanes, Ajay B. Satpute

**Affiliations:** 1 Department of Neuroscience, Pomona College, Claremont, CA USA; 2 Department of Radiology, Athinoula A. Martinos Center for Biomedical Imaging, Massachusetts General Hospital and Harvard Medical School, Boston, MA USA; 3 Department of Clinical and Health Psychology-Serra Húnter Programme, Universitat Autònoma de Barcelona, Barcelona, Spain; 4 Department of Psychology, Northeastern University, Boston, MA USA

**Keywords:** Emotion, Human behaviour

## Abstract

Research on rodents and non-human primates has established the involvement of the superior colliculus in defensive behaviours and visual threat detection. The superior colliculus has been well-studied in humans for its functional roles in saccade and visual processing, but less is known about its involvement in affect. In standard functional MRI studies of the human superior colliculus, it is challenging to discern activity in the superior colliculus from activity in surrounding nuclei such as the periaqueductal gray due to technological and methodological limitations. Employing high-field strength (7 Tesla) fMRI techniques, this study imaged the superior colliculus at high (0.75 mm isotropic) resolution, which enabled isolation of the superior colliculus from other brainstem nuclei. Superior colliculus activation during emotionally aversive image viewing blocks was greater than that during neutral image viewing blocks. These findings suggest that the superior colliculus may play a role in shaping subjective emotional experiences in addition to its visuomotor functions, bridging the gap between affective research on humans and non-human animals.

## Introduction

A midbrain structure in the oculomotor system, the superior colliculus (SC) receives direct retinal input and contains visual neurons with retinotopically organized receptive fields^1,2^. The SC has been proposed to encode a visual saliency map^3^ and contributes to saccadic activity via descending projections to the brainstem^4^. In addition, the SC has also been shown to direct covert visuospatial attention without eye movement^5–7^. The SC is anatomically and functionally segregated into superficial, intermediate, and deep layers^8,9^. Pharmacological and anatomical studies of nonhuman animals have shown that visuosensory and motor layers of the SC are directly linked through bidirectional pathways^10^, and the SC’s intrinsic connectivity is key to its role in visuomotor integration^8,11^.

Although the SC is most commonly characterized by its visuomotor functions^8^, behavioural and pharmacological studies in rodents and non-human primates have demonstrated that it is also implicated in approach and defense behaviours, which are modulated by the nigrotectal connections to substantia nigra^12–14^. In the rodent SC, visual inputs to the lateral and medial parts of intermediate and deep layers are functionally segregated to facilitate approach and defensive behaviours toward appetitive and threatening stimuli, which are typically presented in the lower and upper visual fields respectively^15^. Electrical and chemical stimulation of the rodent SC produces freezing and fleeing behaviours, similar to the response profile of the periaqueductal gray (PAG), an adjacent brainstem nuclei involved in defensive behaviours and fear processing^16,17^. In nonhuman primates, pharmacological activation of the SC through microinfusion triggers reflexive defensive responses, which are partly attenuated by amygdala basolateral complex inhibition^18,19^.

In humans and non-human primates, the SC has been proposed to play a role in rapid visual threat detection, as a part of the SC-pulvinar-amygdala subcortical magnocellular pathway^20–23^. Lesion experiments in infant monkeys have shown that the SC is involved in processing visually identifiable ecological threats^24^. A recent study involving human participants has found greater activity in the SC when snake images are presented in the foveal compared to the peripheral visual field, consistent with the central bias in processing ecologically relevant threat stimuli^25^. In addition, *in vivo* diffusion tensor imaging and probabilistic tractography studies have predicted fiber connections between the human SC and the amygdala via the pulvinar, providing anatomical evidence supporting the SC’s role in threat detection^22,26–28^. SC activity is also influenced by descending projections from the extrastriate cortex, the midtemporal area, and the motor and premotor cortices in the primate brain^8,29^. In both cases, SC activity may be expected to be modulated during affective processing especially when the visual stimulus requires orienting to threat.

At standard fMRI resolution (eg. 2–3 mm isotropic), it is challenging to distinguish the SC from surrounding nuclei such as the PAG. Although there is an abundance of neuroimaging literature on the PAG’s involvement in affective visual processing, evidence from studies of non-human animals suggests that both regions contribute to the activity in response to affective visual stimuli. Most fMRI methods cannot discern PAG response from SC response when viewing aversive vs. neutral images, as standard normalization and smoothing procedures introduce significant partial-volume issues.

**Figure 1:**
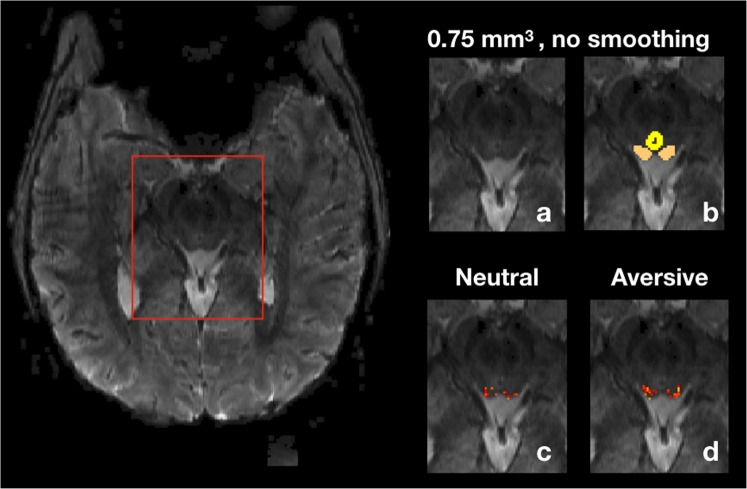
7-T, high-resolution fMRI isolates activity in the SC from surrounding nuclei. A transversal slice from the high-resolution fMRI data without smoothing is shown on the left. In the top row, (panel a) zooms in on the brainstem area; (panel b) shows manual SC and PAG delineations. The SC and the PAG can be clearly discerned at 0.75 mm resolution. (Panels c and d) show activity in the SC during neutral and aversive image viewing blocks respectively (*z* score > 1 for illustrative purposes; analyses were conducted on signal averaged across voxels in each mask).

In this study, we overcame the technical challenges of discerning the SC from surrounding nuclei by using ultra-high field 7 Tesla fMRI. At a nominal isotropic resolution of 0.75 mm, we segmented the SC from functional scans (see Fig. 1) and investigated SC activation while participants viewed a set of natural scene images^30^. To test for visual processing specificity, we compared BOLD signal in the SC with the inferior colliculus (IC), an auditory midbrain region selected for its comparable size and location. We then tested whether activation in the SC was greater during aversive image viewing blocks compared to that in neutral image viewing blocks.

## Results

**Figure 2:**
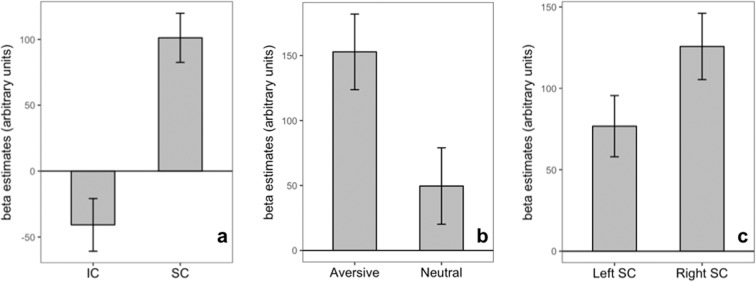
Group-level difference in activation in colliculi regions (panel a), SC activation in response to neutral and aversive visual stimuli (panel b), and SC lateral locations (panel c). The gray bars show the mean activation across subjects, and the error bars show +/−1 standard error of the mean. Both SC showed greater activation in response to visual stimuli compared to the IC (panel a). SC activation was significantly greater in response to aversive visual stimuli than neutral visual stimuli (panel b). Right-ward lateral asymmetry in SC activity was also found (panel c).

We first examined whether visual processing selectively elicited greater activation in the SC compared to a control region, the IC. The goal of this analysis was to provide convergent validation from functional data of our structural localization of the SC insofar as the SC is more intimately related to visual processing than is the IC. Consistent with this notion, the SC had overall greater activity during image viewing blocks than did the IC [paired $t(10)=5.3361$, $p=0.0003$, ${d}_{z}=1.6089$] (Fig. 2, panel a).

Next, we investigated whether activity in the SC was further modulated by affective processing. The SC showed, as predicted, greater activation during aversive image viewing blocks compared to neutral image viewing blocks [paired $t(10)=2.2889$, $p=0.0451$, ${d}_{z}=0.6901$] (Fig. 2, panel b). As noted in the Methods, image features including contrast, luminance, and complexity did not strongly vary between the aversive and neutral categories of images. Nevertheless, we repeated our analysis after controlling for these features by including them as regressors in the first-level general linear models. The significance level was marginally reduced, but the estimated effect size remained high [paired $t(10)=2.1351$, $p=0.05851$, ${d}_{z}=0.6438$].

Although our primary hypotheses concerned the SC and image category, we additionally report findings for laterality effects during overall image viewing. For lateral asymmetry in SC, prior work suggests that laterality effects in the SC may occur due to ocular dominance. A within-subject 2x2 ANOVA model across stimulus categories and lateral locations revealed a main effect of laterality, where the right SC was more strongly engaged during visual processing overall compared to the left SC [$F(1,10)=16.505$, $p=0.002$, ${\eta }_{p}^{2}=0.623$] (Fig. 2, panel c). We also examined the interaction effect between laterality and image category, but it did not reach significance [$F(1,10)=0.527$, $p=0.484$, ${\eta }_{p}^{2}=0.050$]. Intriguingly, the laterality effect was attenuated when image property regressors were added to the first level general linear models [$F(1,10)=0.471$, $p=0.508$, ${\eta }_{p}^{2}=0.045$], suggesting that lateral differences in SC activity may be driven by visual features of the images.

## Discussion

In this study, we examined functional activity in the human SC during affective visual stimulus processing. Functional activity in the SC was localized with high fidelity using ultra high-field, high-resolution fMRI and a custom segmentation procedure. The precise anatomical segmentation of the SC converged with functional results showing greater SC activity during visual stimulus processing in comparison to a similarly sized and spatially proximal control region, the IC. Supporting our main hypothesis, we then tested for and found greater functional activity in the SC during affective image viewing in comparison to neutral image viewing.

The present findings have important implications for fMRI studies of affective processing in the human midbrain. Apart from a few notable exceptions^20,31,32^, the SC is often overlooked in the human affective neuroscience literature. Instead, the majority of this work has focused on the adjacent PAG^33–37^. However, both regions have been implicated in defensive behaviour based on research in non-human animals^16,17,38–40^. The present findings in combination with our prior report^35^ suggest that affective image processing also engages both regions. In this respect, our findings underscore the importance of ultra high-field neuroimaging techniques for overcoming partial volume effects and examining the functional role of specific brainstem nuclei in affective processing^41^.

Previous neuroimaging studies have shown that midbrain activation in the vicinity of the SC is modulated by negative affective content, such as affective facial expression stimuli, independent of eye movement or covert attention^42–44^. Presentation of faces that were conditioned to predict pain elicits greater activity in the vicinity of SC compared to presentation of unconditioned faces even in the blind hemifield of a cortically blind patient^45^. However, previous studies were also limited insofar as the techniques are unable to draw firm conclusions about the SC given the larger voxel resolutions (>2 mm isotropic) and partial volume effects introduced by smoothing (>6 mm FWHM). Although recent reviews of affective modulation of visual perception^20,31,32^ allude to the SC as a part of the affective visual system, our study demonstrates for the first time that the human SC is engaged in processing negative affective content in visual stimuli that signal threat or harm, which are of great ecological importance to humans and non-human animals.

The SC has been extensively studied as an oculomotor region important for defensive responding^10^. Findings in rodents, for example, have uncovered the importance of the SC in defensive responding using electrophysiological^16,17,46^, pharmacological^12,16,19,47^, and optogenetic^13,14,46,48^ methods. Studies in non-human primates have generalized the SC’s role in defensive behaviour and visual threat detection of natural predator using pharmacological^18^ and lesion^24^ methods, respectively. Due to structural and functional differences in the SC across mammalian species^8^, it is important to investigate the SC in humans. This study provides a methodology for bridging studies of defensive behaviours in non-human animals with neuroimaging research of human affective experience.

We^49^ and others^50–53^ have proposed that affective experience relies on a distributed neural architecture that includes functional activity in early sensory systems. Our findings suggest that for visual affective processing, this model may be extended to include subcortical structures such as the SC. Of interest is whether the effects observed here resulted from the SC being part of an ascending SC-pulvinar-amygdala visual pathway^20,22,25–27^ or rather from descending (top-down) input from distributed cortical areas (for a review of cortical input to the SC, see^8^).

While a number of neuroimaging and lesion studies have provided support for the functional role of the SC in affective visual processing through the SC-pulvinar-amygdala subcortical pathway^23,54^, there has also been anatomical evidence from studies of monkey brains against the existence of such a subcortical pathway in primates (for a review, see^55^). The SC may facilitate responding to threat by rapidly identifying context-salient visual features (e.g. threats arriving from different locations in the environment) and prepare the rapid coordination of behaviours accordingly (fleeing from or orienting toward these locations), with descending corticotectal pathways providing information about which contexts and features are more or less likely to be of relevance. Connections between the SC and the PAG are known to support these behaviours^19^. Notably, other fMRI studies have shown greater neural activity in midbrain regions in humans during increasing threat proximity^34^ and also during simulated gun shooting decisions^56^. Future work using high-resolution imaging combined with functional connectivity analyses may help provide insight on the pathways driving functional activity in the SC in affective processing, and further examine the dynamic between SC and PAG during threat processing.

Although not the main focus of the present study, we also observed lateral asymmetry in the SC’s response during visual stimulus processing in general. The right SC showed greater activation compared to the left SC, which is consistent with findings from some previous fMRI studies. The lateral asymmetry effect was attenuated after controlling for stimuli visual features such as complexity, luminance, and contrast, suggesting that the observed greater activity in the right SC may be attributed to the processing of these visual features.

While our findings provide support for a role of the human SC in affective visual processing, they also raise several questions for future work. Recent theoretical work has proposed an account of oculomotor behavior based on active inference^57^. As an interface between perception and action with laminated internal architecture and multimodal sensory integration, the SC is an ideal site for investigating feedback and feedforward information flow in a hierarchical predictive coding framework^58^. The present study provides a methodology for isolating functional activity in the SC using high-resolution fMRI. Future work may examine whether the affective modulation of visual stimuli has predictive value for oculomotor behaviour.

## Methods

### Participants

In a prior report, we examined functional activity in the PAG during affective image processing^35^. The present report makes use of the same subject sample and dataset but addresses activation in the colliculi. Thirteen healthy, right-handed volunteers participated in the study and provided informed consent. Individuals received compensation for their participation. The study was conducted in accordance with the guidelines of the Partner’s Health Institutional Review Board, which approved all procedures. Two participants were excluded due to a hard drive failure (1 subject) and ghosting (1 subject). In the remaining subjects, one participant only has data from the first two runs out of a total of three runs because of a failure in the stimulus display computer. Functional data collected from 11 participants (five male, age range, 20–35 y) were included in the analysed sample.

### Experimental design and behavioural analysis

Participants viewed a sample of 30 highly emotionally aversive photographs and 30 neutral photographs from a database of images normed to elicit affective experiences^30^. Each block consisted of five images of one category, randomly sampled. Each image was presented for 2 s, and the inter-stimulus intervals ranged from 0.5, 1, 1.5, 2, to 2.5 s. One block of image presentation lasted 17.5 s in total. After each image viewing block, participants were prompted to report their experience across five categories, “Activated” (for arousal), “Angry”, “Disgusted”, “Sad”, and “Scared”, using a five-button response box. The labels were presented sequentially and in a random order with numbered scales from 0–4 to indicate the amount of affect or emotion from none to high. Reports were obtained during a 16 second period before the start of the next image viewing block. The self-report measures assessed the valence and emotional intensity of the aversive images, indicating that subjects experienced more anger [${t}_{robust}(9)=8.86$, $P < 0.00001$], disgust [${t}_{robust}(10)=5.74$, $P < 0.001$], sadness [${t}_{robust}(10)=3.96$, $P < 0.01$], and fear [${t}_{robust}(10)=4.60$, $P < 0.001$], but their self-reported levels of arousal did not increase significantly [activated; ${t}_{robust}(10)=0.22$, $P < 0.84$]. Image visual properties were extracted from neutral and aversive stimuli (described below) and no statistically significant difference was found between the complexity [$p=0.859$], contrast [$p=0.720$], or luminance [$p=0.055$] across stimuli categories. Some blocks of images sampled from another stimulus database (for details: K. Kveraga http://nmr.mgh.harvard.edu/kestas/affcon) were included for exploratory purposes, but they were not the focus of the analyses in the present work.

### Functional MRI acquisition

BOLD-fMRI images were acquired on a 7 Tesla Siemens MRI scanner (Siemens Healthcare). We used a whole body gradient-coil (SC72AB) with maximum gradient amplitude of 70 mT/m, and maximum slew rate of 200 mT/m/ms. Functional MRI images were collected using single-shot gradient-echo EPI with the following parameters: TR = 3000 ms; TE = 26 ms; flip angle (FA) = 90°; 40 contiguous slices with oblique axial/coronal orientation, approximately perpendicular to the aqueduct; nominal voxel resolution = 0.75 mm^3^; field of view (FOV) = $192\times \,192\,{{\rm{m}}{\rm{m}}}^{2}$; number of repetitions = 90; GRAPPA acceleration factor = 4; echo spacing = 1.04 ms, effective echo spacing = 0.26 ms; bandwidth = 1,148 Hz per pixel; partial Fourier in the phase encode direction: 6/8.

### Functional MRI preprocessing and analysis

Functional images were preprocessed with the FMRIB Software Library (FSL)^59–61^. Preprocessing steps were performed on each functional run separately. Functional time-series data were motion corrected to the middle slice within each run and filtered using a high-pass temporal filter with cut-off frequency equal to 0.01 Hz. We applied a 6-parameter rigid-body transform using the MCFLIRT tool in FSL. The maximal relative displacement ranged between 0.15 to 1.45 mm across scans (median = 0.39 mm, interquartile range = 0.29–0.61 mm). Similar to the procedures in our previous work^35^, no smoothing or normalization was performed at this stage.

Next, we isolated the SC and the IC (4 masks) for each functional run by manual segmentation. Masks were hand drawn using FSLeyes directly from the functional data. Because of their comparable size and adjacent anatomical location to those of the SC, the IC were selected as control regions to investigate the specificity of SC activity in response to visual stimulus processing. The masks were produced based on anatomical markers that delineate the SC and the IC from surrounding tissue^62^. The medial and posterior boundaries are demarcated respectively by the positions of the PAG^35^ and of the cerebral spinal fluid identified on a region of high signal variability (see Fig. 1 panel b and Supplementary Fig. S1). We then confirmed the shapes of the ROIs based on high-resolution FLASH images for each subject as well as high-resolution structural images of the SC at 9.4 Tesla MRI from an anatomical atlas^63^.

For our main statistical comparisons, we first averaged the time course signal across voxels in the SC and the IC to maximize signal-to-noise ratio, then applied general linear models consisting of regressors for stimulus onsets by image type convolved with the double gamma hemodynamic response function, their first-order temporal derivatives, and motion regressors to obtain parameter estimates for neutral and aversive conditions. Although we did not focus on voxel-wise analysis, we have provided a figure for illustrative purposes showing voxels with *z* > 1 in the SC and the IC for aversive and neutral conditions at the functional resolution of 0.75 mm^3^ (see Fig. 1 panels c,d and Supplementary Fig. S2). We used robust regression^64^ to minimize the influence of outliers. Motion parameters were included in the model to account for motion-induced response fluctuations. The parameter estimates for left and right SC and IC under aversive and neutral conditions were submitted to two-tailed paired t tests to examine the main effects of interest. Cohen’s ${d}_{z}=t/\sqrt{n}$ was used to calculate t test effect sizes given our within-subject design^65^. A 2 × 2 ANOVA across SC lateral locations and stimulus condition was used to examine lateral asymmetry effects. In a second analysis, regressors of image visual properties (complexity, contrast, and luminance) were added to the general linear model to control for their influence on SC activity. Image visual properties, including complexity, contrast, and luminance, were computed using a custom MATLAB script. Image complexity was measured by edge density, which is the ratio of edge pixels identified by MATLAB’s canny edge detector to non-edge pixels after the RBG image has been converted to gray scale^66^. Image contrast was measured by the maximum intensity minus the minimum intensity of the converted gray-scale image. Image luminance was the mean luminance of all pixels after converting the image to HSV format, which separates image intensity from color information.

## Supplementary information

Supplementary information.

## Data Availability

The fMRI datasets generated during and/or analysed during the current study are available from the corresponding author upon reasonable request. The script for the main statistical analysis is available on Github: https://github.com/candiceyuxiwang/7TSC.
