# Supplementary material for: Ultra High Field fMRI of Human Superior Colliculi Activity during Affective Visual Processing
Source: Sci Rep. 2020 Jan 28;10:1331. doi: 10.1038/s41598-020-57653-z (PMC6987103; doi:10.1038/s41598-020-57653-z)
Supplement: Supplementary file 1 — Supplementary information. [file 41598_2020_57653_MOESM1_ESM.docx]

**Supplementary Information**

Ultra High Field fMRI of Human Superior Colliculi Activity during Affective Visual Processing

Authors: Yuxi C. Wang, Marta Bianciardi, Lorena Chanes, Ajay B. Satpute


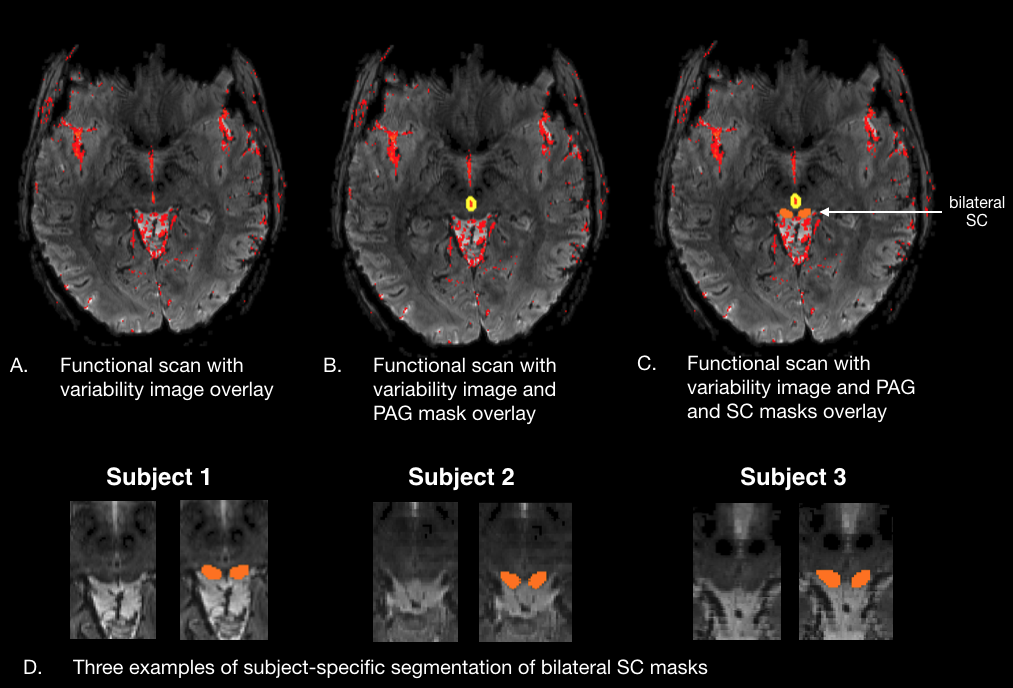


**Figure S1.** Superior colliculi masks. Subject-specific SC masks were produced using anatomical markers in the functional data that delineate posterior and anterior boundaries (Loureiro et al., 2016). Signal variability image (panel A) and PAG mask (panel B) from a previous study (Satpute et al., 2013) were used for reference in determining the precise location of SC relative to cerebrospinal fluid and the PAG in a given subject (panel C). The SC’s shape and relative location to nearby nuclei are referenced using a human brainstem anatomy atlas (Naidich et al., 2009). Panel D shows three examples of subject-specific SC masks drawn directly on functional data.

Subject 1 SC Aversive Neutral


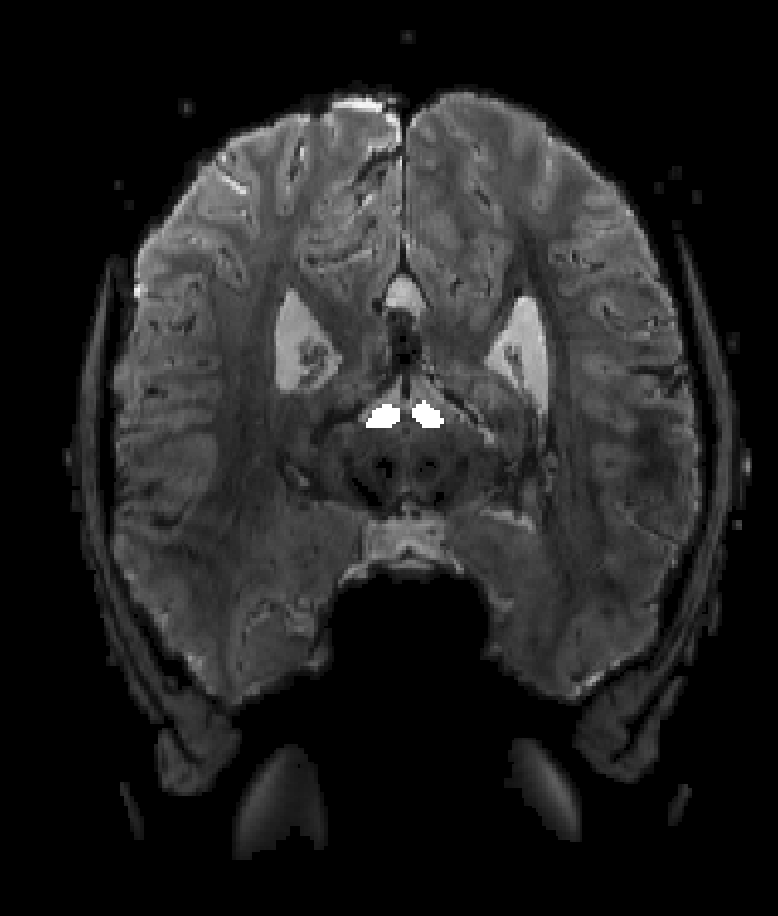

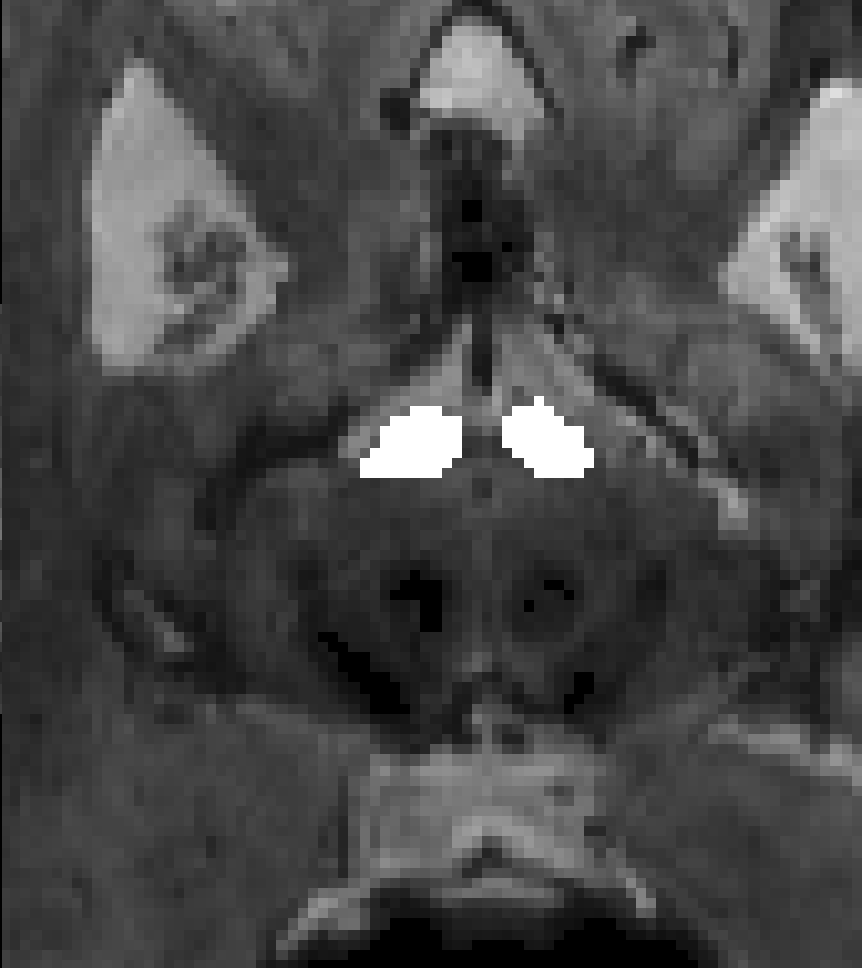

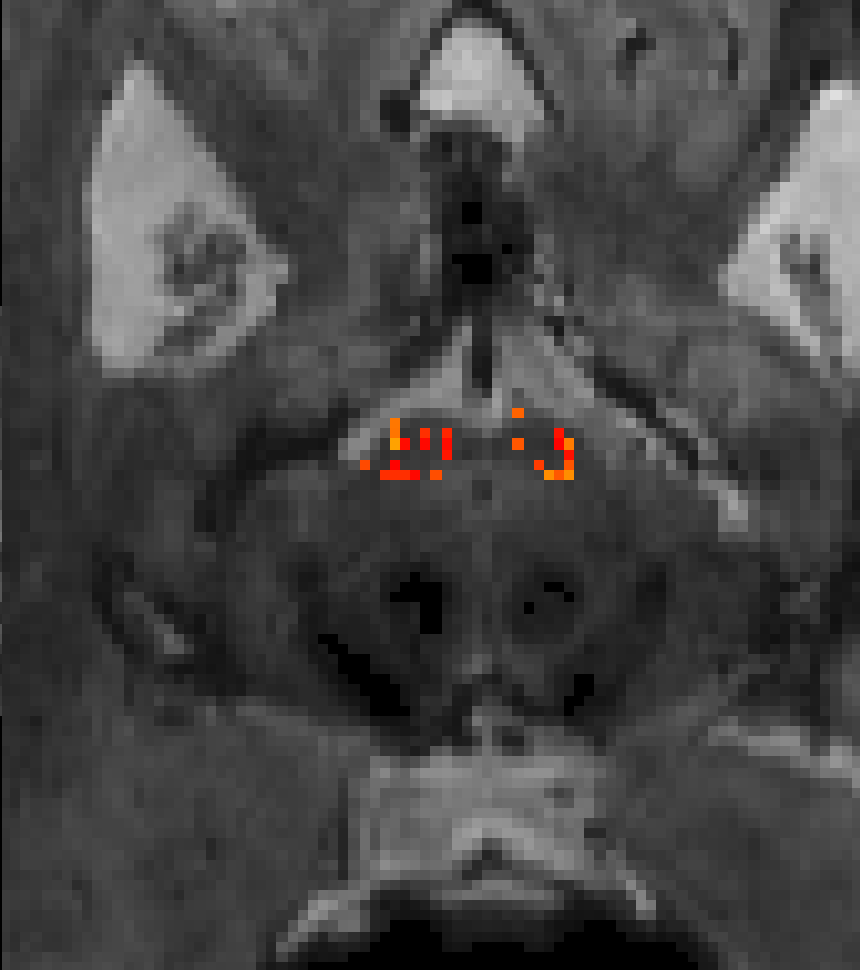

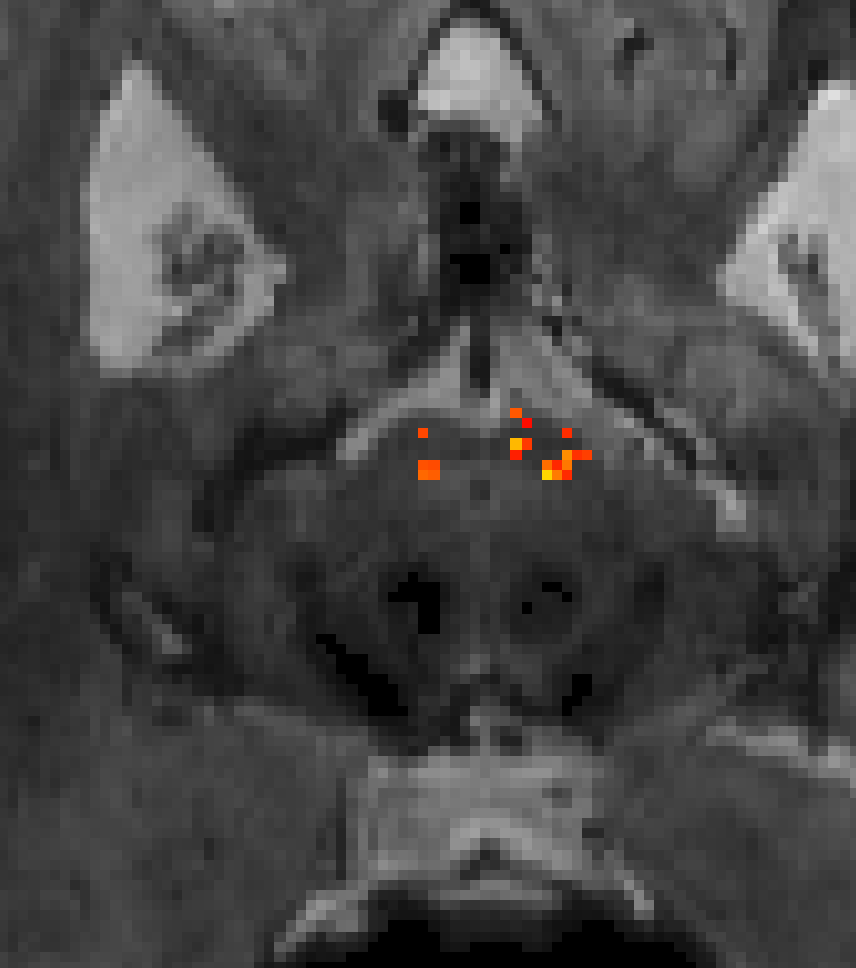


C

B

A

IC Aversive Neutral


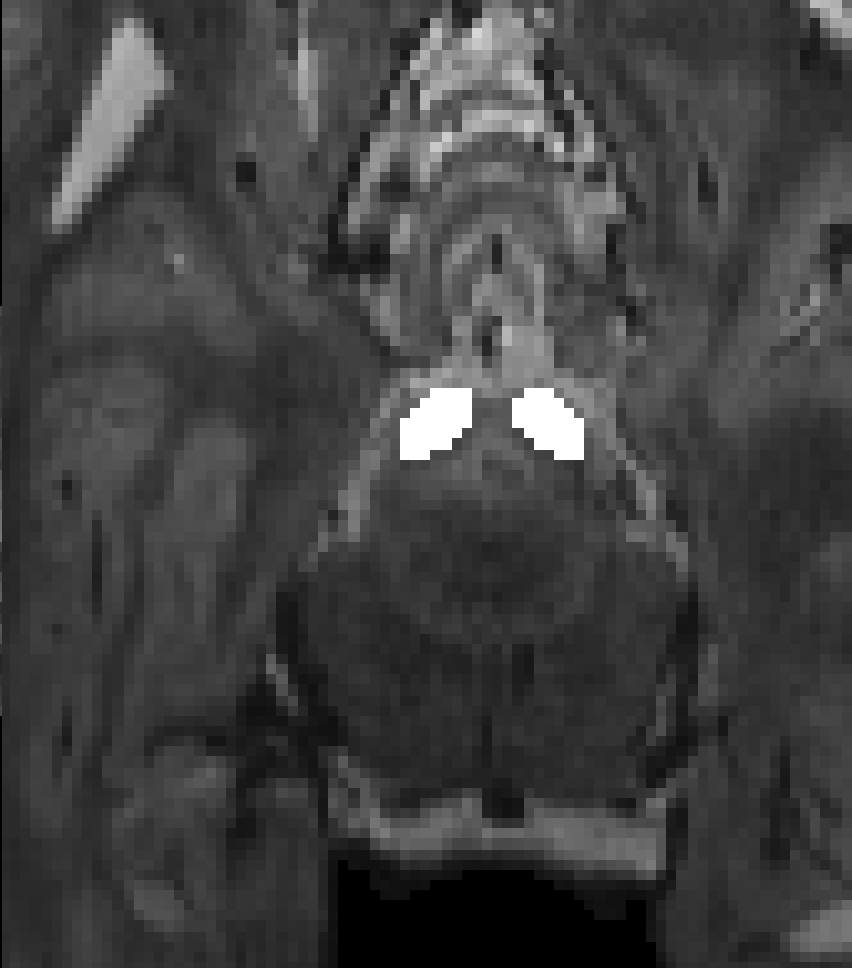

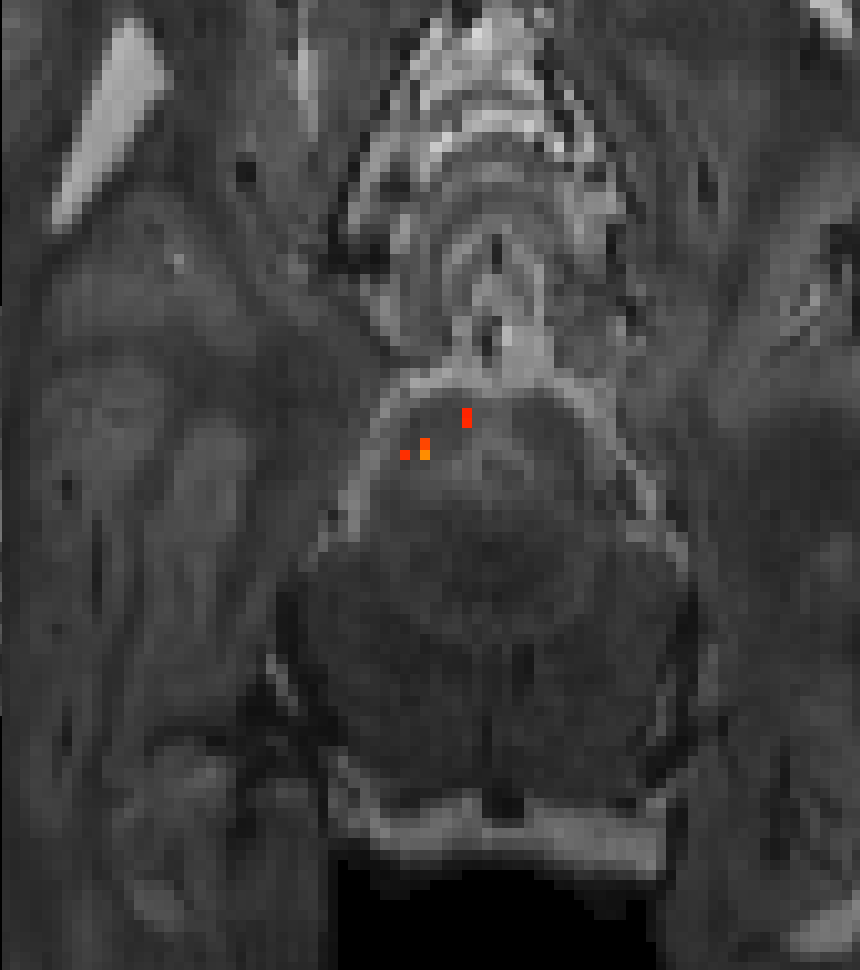

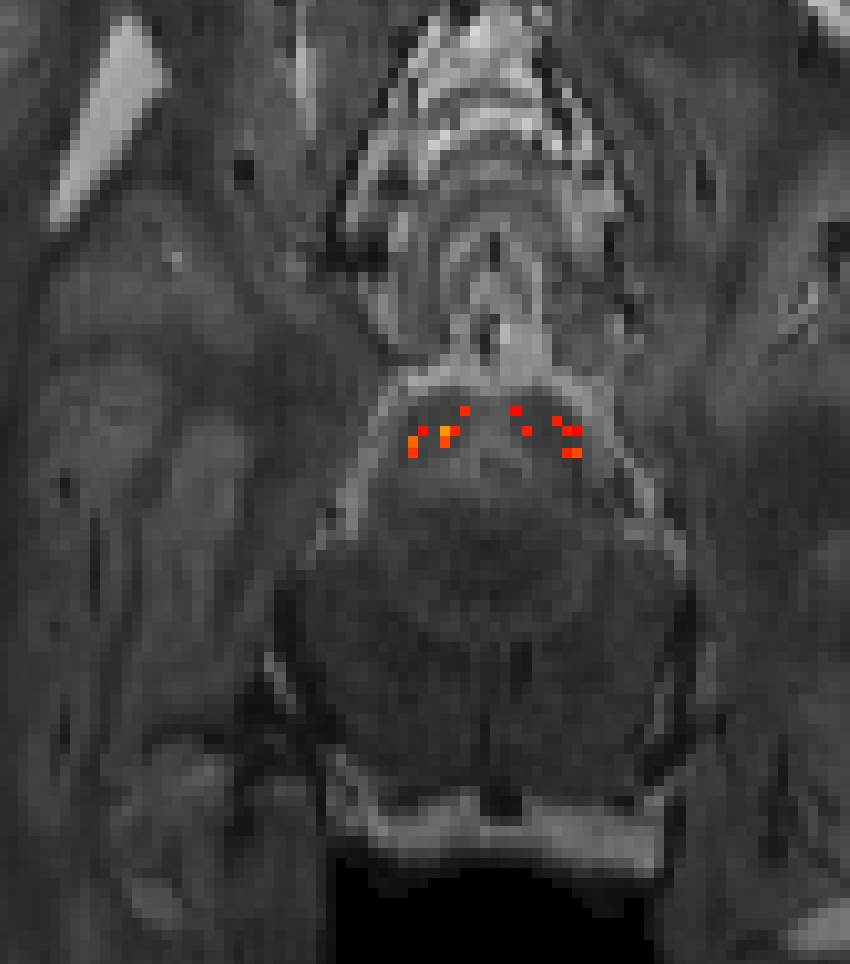


F

E

D


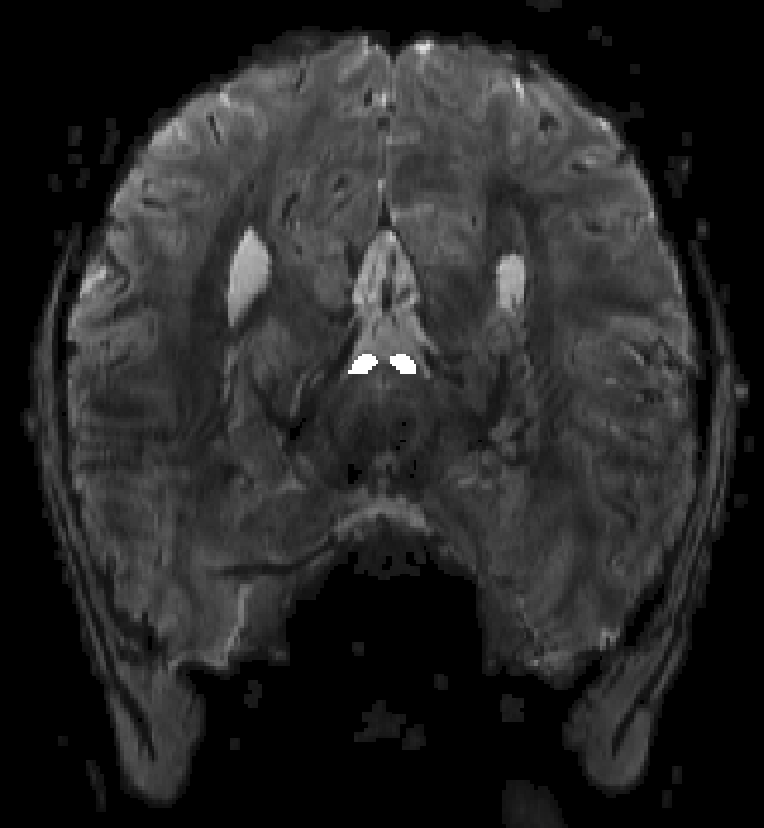
 Subject 2 SC Aversive Neutral


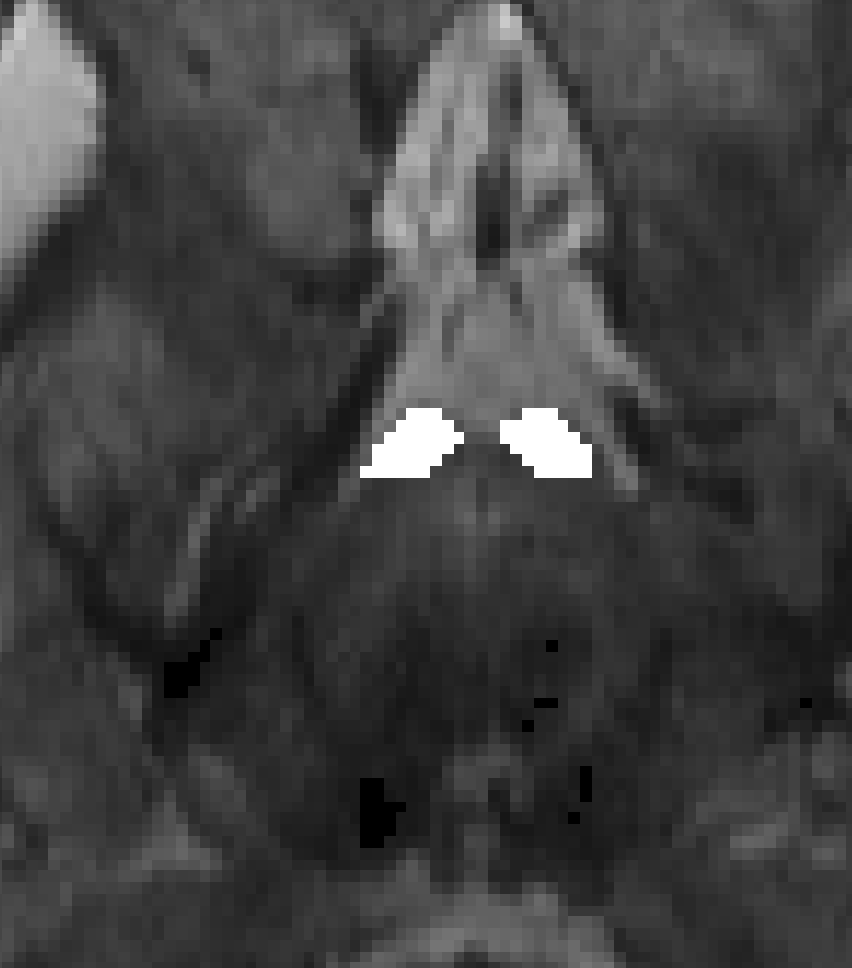

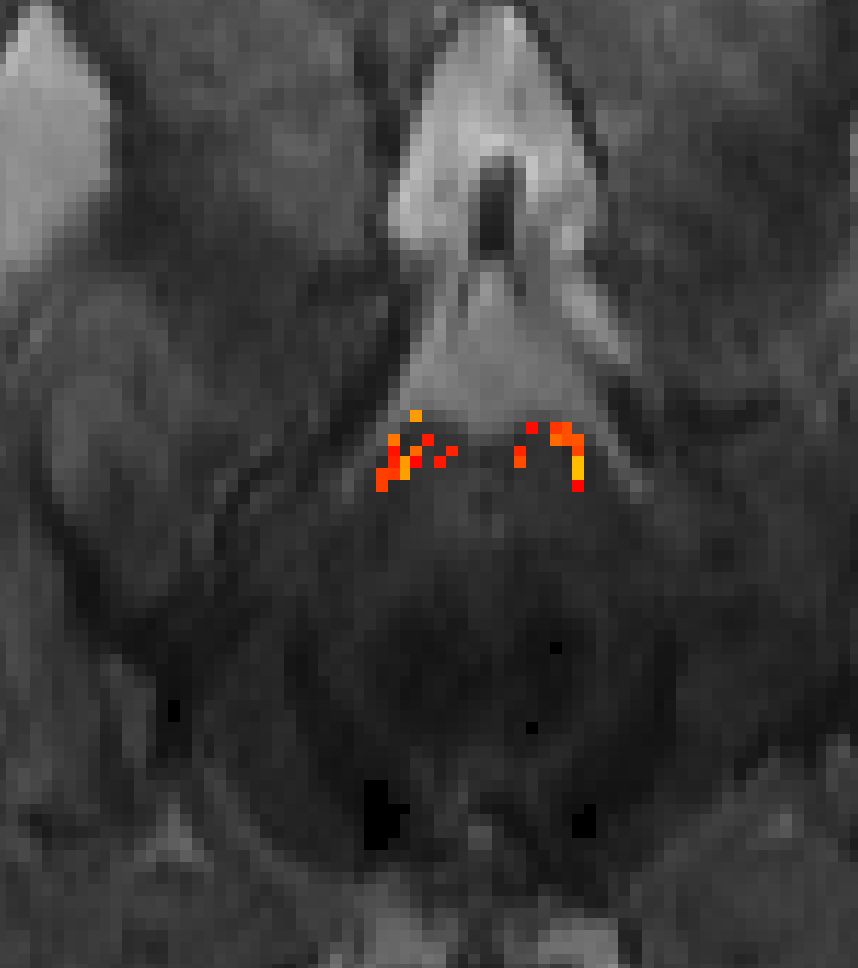

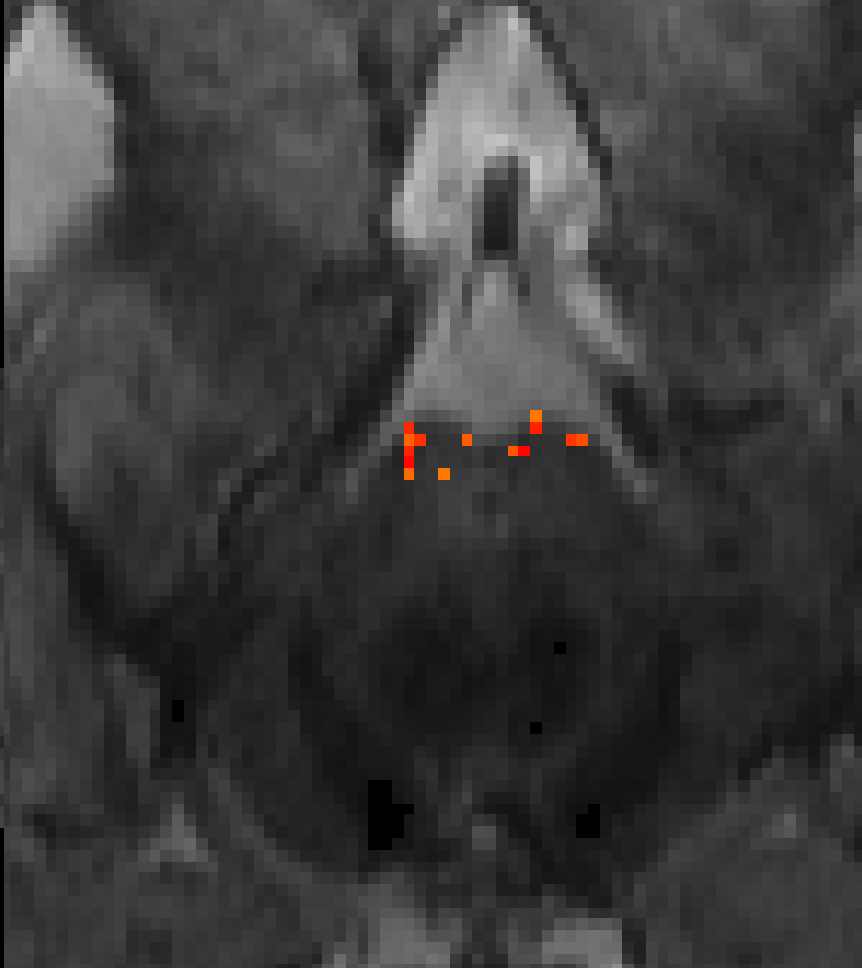


IC Aversive Neutral


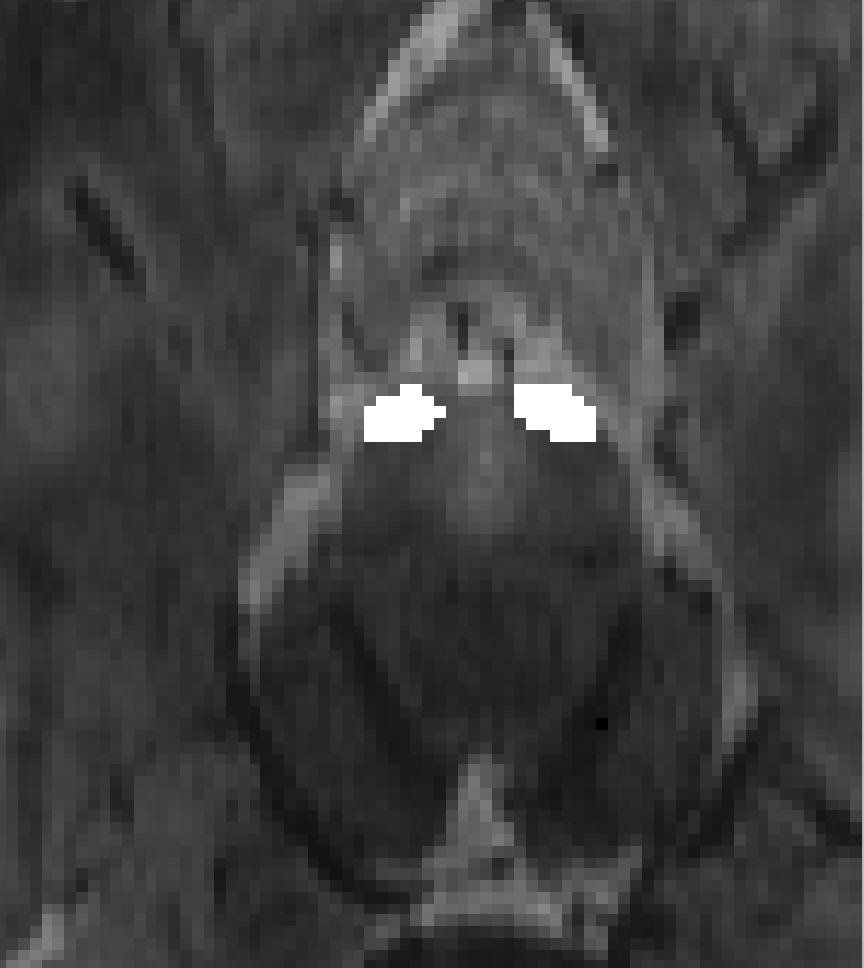

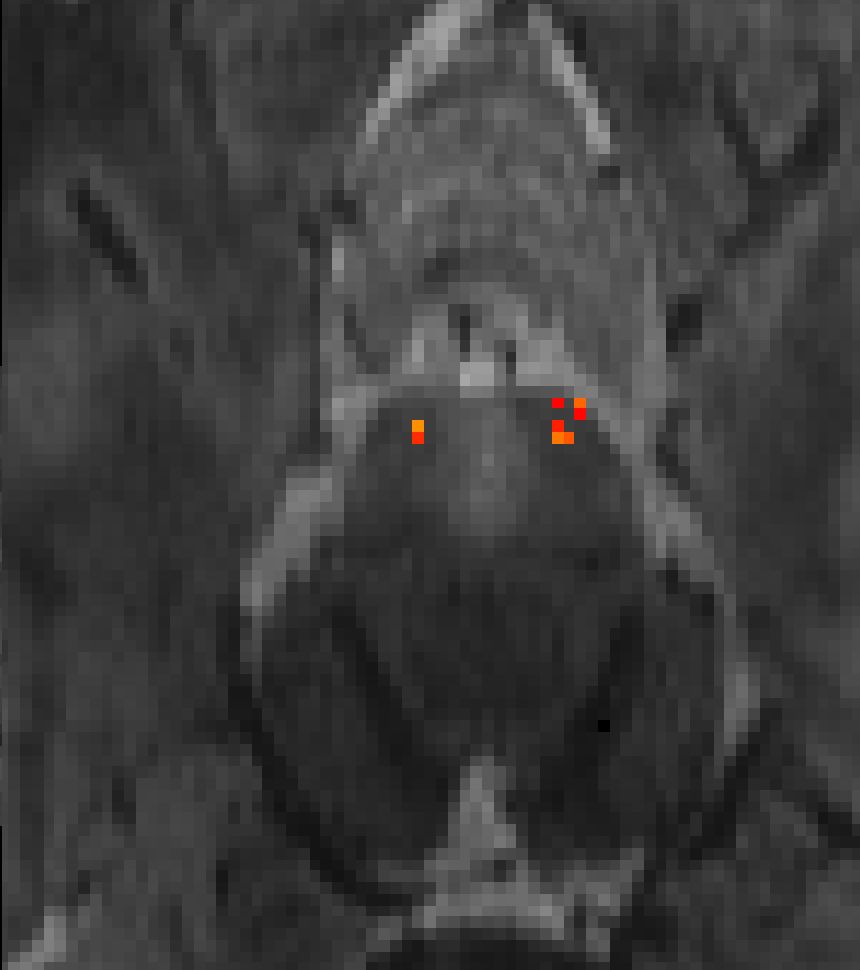

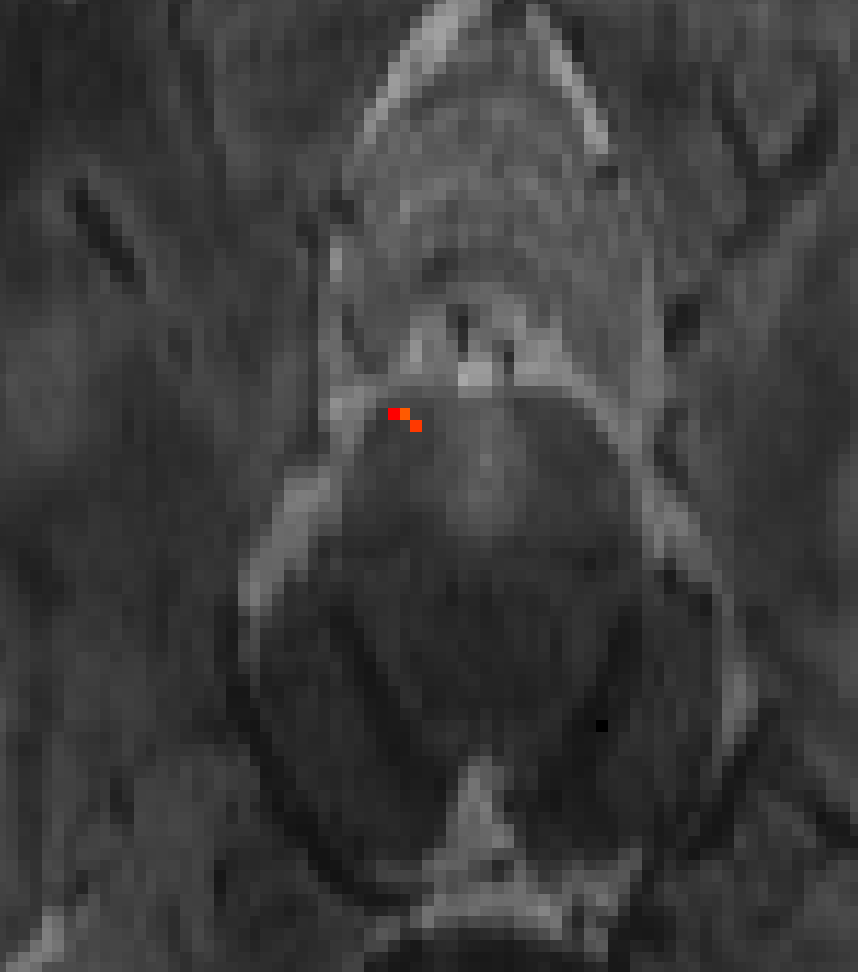


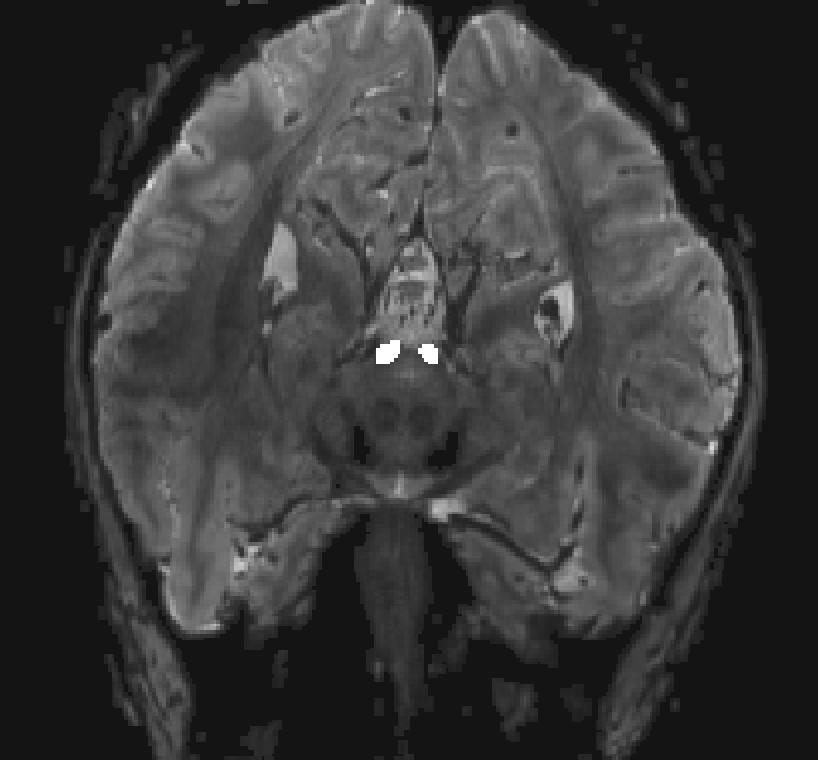
 Subject 3 SC Aversive Neutral


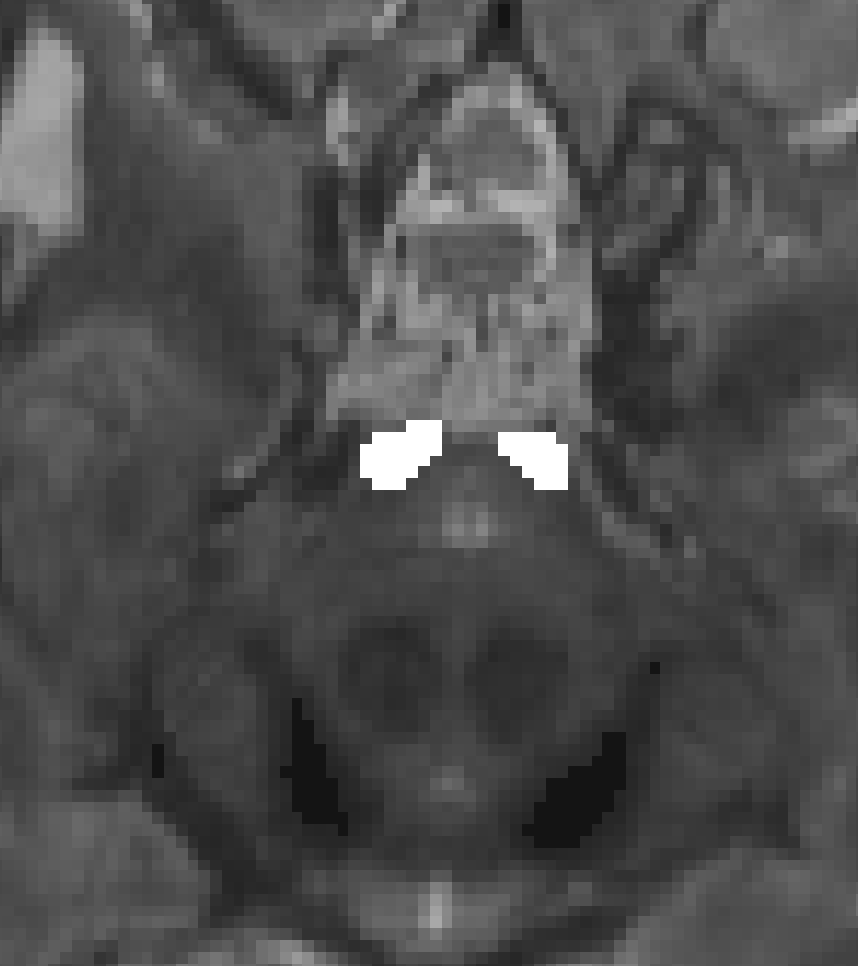

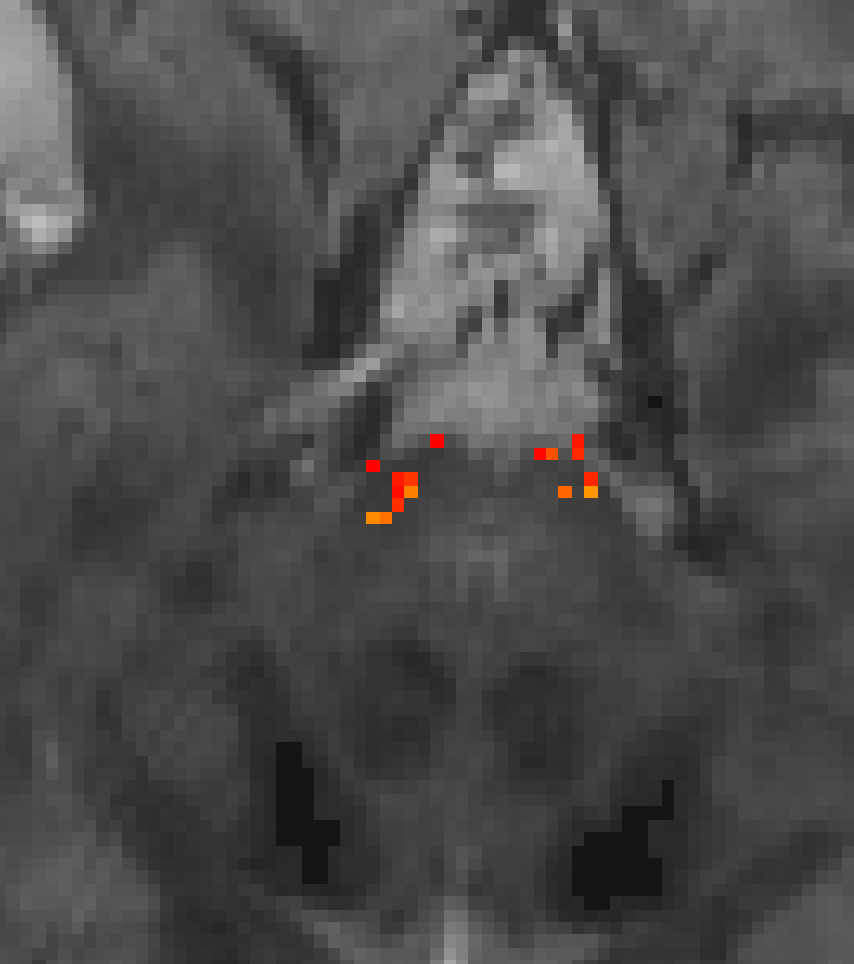

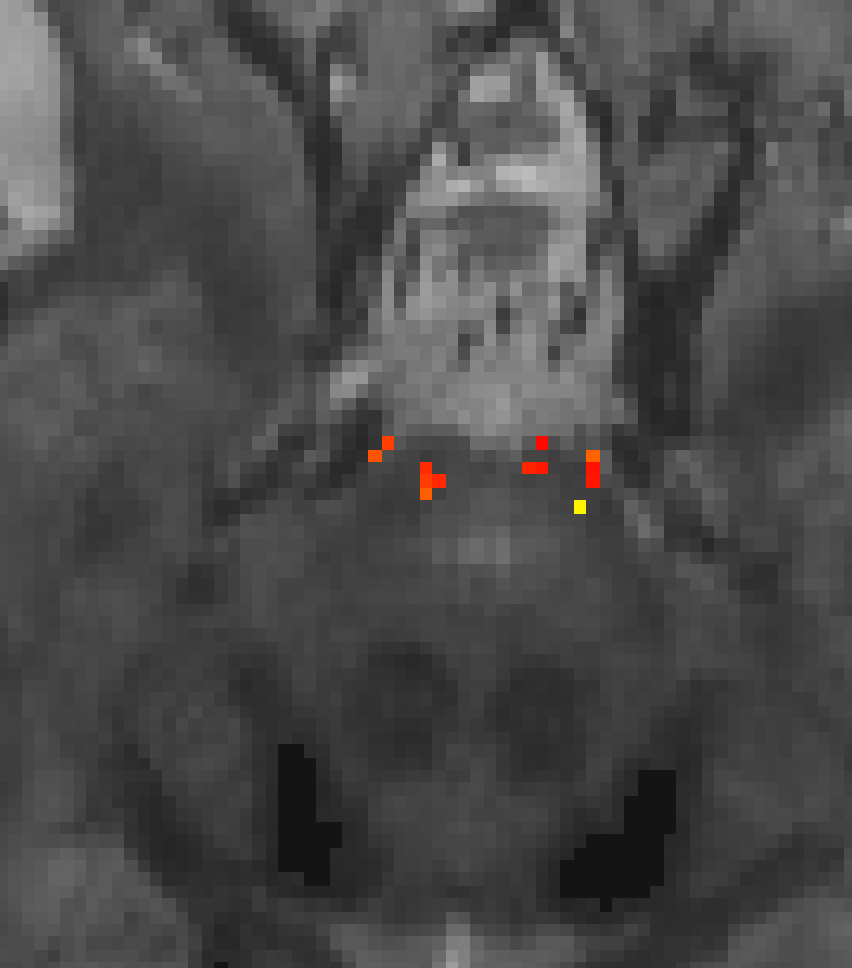


IC Aversive Neutral


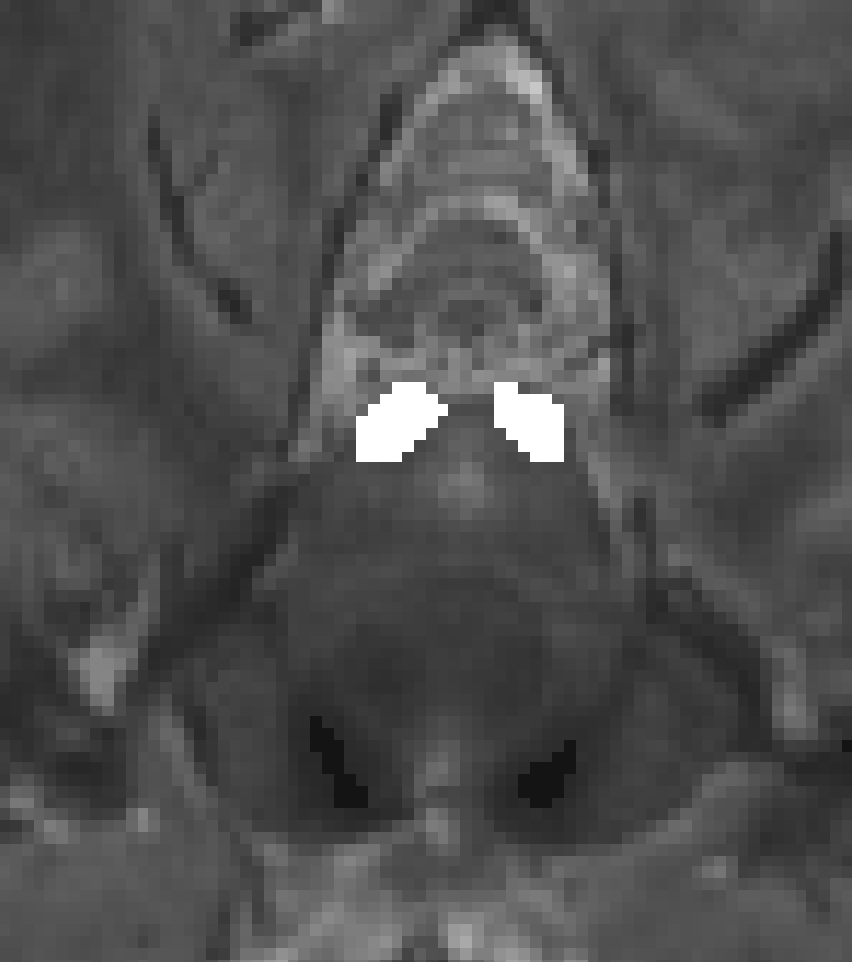

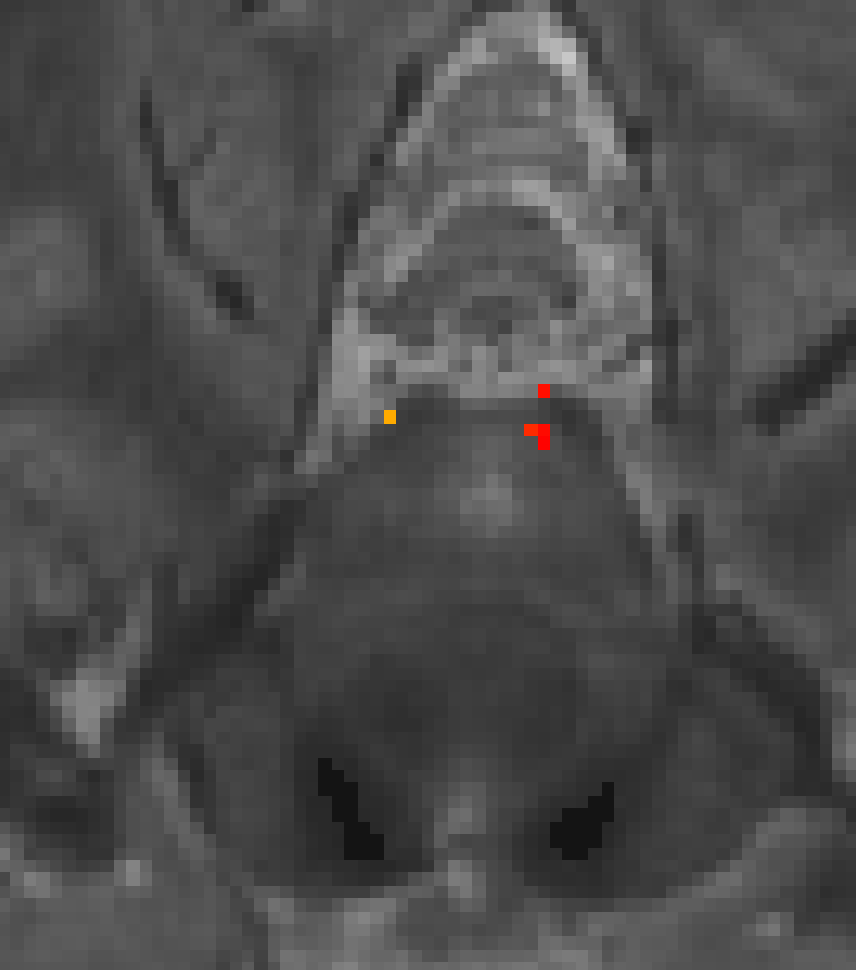

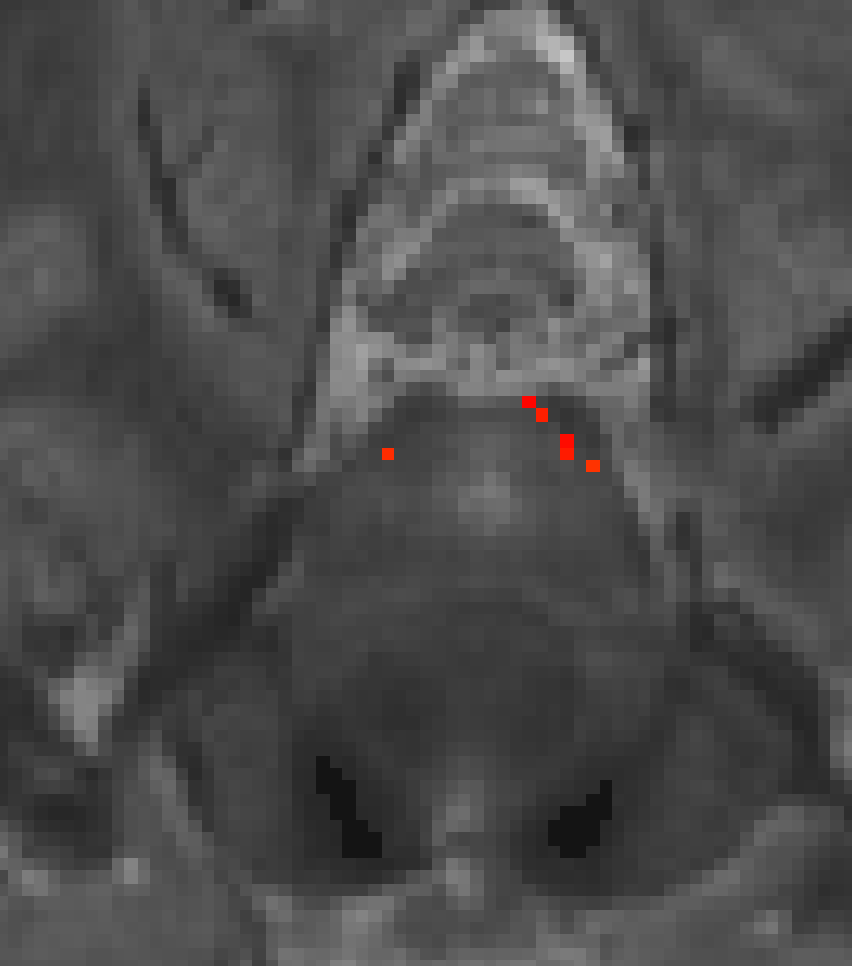


**Figure S2.** Voxel-wise functional activity in superior colliculi during aversive and neutral image viewing for 3 subjects. In the analyses reported in the main text, the time series signal was averaged across all voxels within each mask prior to performing statistical analyses in order to maximize signal-to-noise. It is possible that voxels driving the effect are primarily localized to areas bordering the PAG, however, as shown by this figure that does not seem to be the case. The supplementary figure illustrates uncorrected statistical maps when performed on the time series for each voxel separately (Z > 1 for the comparisons of regressors corresponding to aversive images minus baseline and neutral images minus baseline). Voxels spread across the SC appeared to have nominally greater activity during image viewing blocks. The IC also shows activity in some, but generally fewer, voxels during these conditions. Panels A and D show custom masks for superior and inferior colliculi respectively, overlayed on the mean functional image in transversal view. Panels B and C show the voxels with nominally greater activity in the superior colliculi during aversive and neutral image viewing blocks, respectively (Z > 1). Panels E and F show the voxels with nominally greater activity in the inferior colliculi during aversive and neutral image viewing blocks respectively (Z > 1).
